# Comparison of Different Timing of Multivessel Intervention During Index-Hospitalization for Patients With Acute Myocardial Infarction

**DOI:** 10.3389/fcvm.2021.639750

**Published:** 2021-06-10

**Authors:** En-Shao Liu, Cheng Chung Hung, Cheng-Hung Chiang, Chia-His Chang, Chin-Chang Cheng, Feng-You Kuo, Guang-Yuan Mar, Wei-Chun Huang

**Affiliations:** ^1^Department of Critical Care Medicine, Kaohsiung Veterans General Hospital, Kaohsiung, Taiwan; ^2^School of Medicine, National Yang-Ming University, Taipei, Taiwan; ^3^Department of Physical Therapy, Fooyin University, Kaohsiung, Taiwan; ^4^Graduate Institute of Clinical Medicine, Kaohsiung Medical University, Kaohsiung, Taiwan

**Keywords:** acute myocardial infarction, multivessel percutaneous coronary intervention, non-ST elevation myocardial infarction, non-infarct-related artery, percutaneous coronary intervention, ST-segment elevation myocardial infarction

## Abstract

**Background:** Many patients presenting with acute myocardial infarction (AMI) were found to have a multivessel disease. Uncertainty still exists in the optimal revascularization strategy in AMI patients. The purpose of this study was to assess the outcome of immediate multivessel revascularization compared with staged multivessel percutaneous coronary intervention (PCI) in patients with AMI.

**Method:** This was a nationwide cohort study of 186,112 patients first diagnosed with AMI, 78,699 of whom received PCI for revascularization. Patients who received repetitive PCI during the index hospitalization were referred to as staged multivessel PCI. Immediate multivessel PCI was defined as patients with two-vessel PCI or three-vessel PCI during the index procedure. Cox proportional hazards regression models were performed to evaluate the different indicators of mortality risks in AMI.

**Result:** Immediate multivessel PCI was associated with a worse long-term outcome than staged multivessel PCI during the index admission (log-rank *P* < 0.001). There was a higher incidence of stroke in patients with multivessel PCI during hospitalization. In Cox analysis, immediate multivessel PCI was an independent risk factor for mortality compared to those with staged multivessel PCI, regardless of the type of myocardial infarction.

**Conclusion:** This study demonstrated that performing immediate multivessel PCI for AMI may lead to worse long-term survival than staged multivessel PCI. Our findings emphasized the importance of PCI timing for non-infarct-related artery stenosis and provided information to supplement current evidence.

## Introduction

Up to 50% of patients with acute myocardial infarction (AMI) undergoing primary percutaneous coronary intervention (PCI) have the multivessel disease (1, 2), which was independently associated with worse clinical outcomes (1, 3). The optimal strategy for treating the non-culprit artery in patients with AMI and multivessel disease remains an unresolved issue. The intervention options include (1) culprit vessel-only PCI with continuous medical treatment and repeat PCI of non-infarct arteries only if recurrent angina or myocardial ischemia on stress testing; (2) culprit vessel-only PCI, followed by staged PCI of non-infarct arteries later during the index admission or soon after discharge; and (3) multivessel PCI at the time of index intervention.

Some theoretical arguments support the complete revascularization of all coronary arteries during the index PCI. The most important benefit is the potential to improve overall myocardial perfusion and function in the acute phase (4–6). However, a multivessel PCI strategy might also pose additional risks, including contrast-induced acute kidney injury, volume overload, prolonged procedure time, and further ischemia (7, 8). Current evidence does not support routine immediate multivessel PCI in the AMI patients with cardiogenic shock and multivessel disease (9). For the remaining AMI patients with multivessel disease but no cardiogenic shock, the timing of intervention for non-culprit lesions is still an unanswered issue (10–16). Therefore, this nationwide cohort study aimed to evaluate the impact of the different timing of multivessel PCI on survival in patients after first AMI.

## Method

### Data Source

The unweighted data was retracted from the National Health Insurance Research Database (NHIRD) from January 2000 to December 2000 to 2012. The National Health Insurance (NHI) in Taiwan is a mandatory health insurance program established in 1995 and covers nearly 100% of Taiwan's population.

The NHIRD contains comprehensive medical records of patients, including inpatient records of demographic information, encrypted de-identification numbers, sex, birth dates, admission dates, International Classification of Diseases, Ninth Revision, Clinical Modification (ICD-9-CM) diagnostic codes, drug codes, and procedure codes. It has been extensively used in epidemiologic studies in Taiwan. The Human Research Committee of Kaohsiung Veterans General Hospital approved this study.

### Definition of AMI Population

The AMI cohort with a total of 186,326 patients was retrieved from NHIRD by the inclusion criteria of patients first hospitalized with a primary diagnosis of AMI (ICD: 410~410.92) between January of 2000 and December of 2012 in Taiwan.

**Figure 1:**
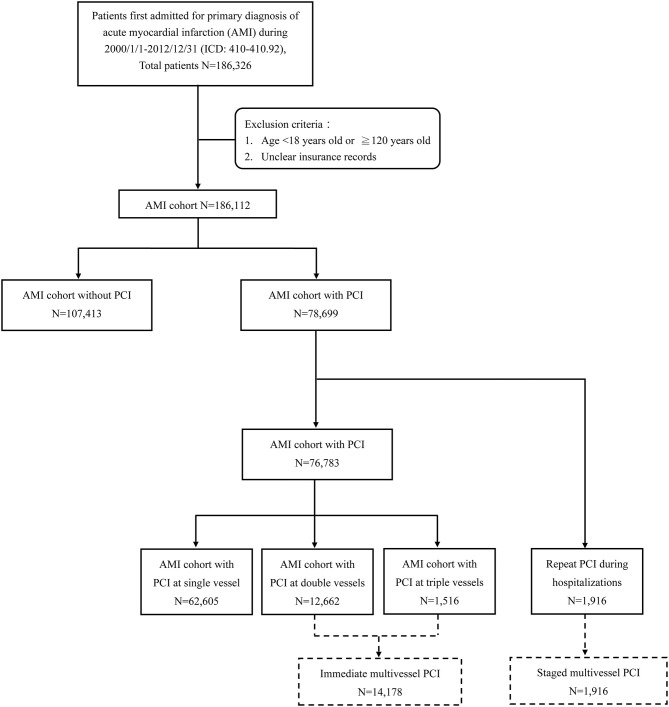
Flowchart of case selection from the Taiwan National Health Insurance Research Database. AMI, acute myocardial infarction; PCI, percutaneous coronary intervention.

Patients whose insurance record was unclear, who were younger than 18 years old, or who were older than 120 years old were excluded from this study. Finally, a total of 186,112 patients were included in the analysis (Figure 1).

### Study Population

Those receiving PCI treatment were identified among patients who were certified with the first hospitalization for AMI. Of the 78,699 cases, the 1,916 patients who repeated PCI during the index hospitalization were selected as the group of staged multivessel PCI. We divided the other 76,783 cases into three groups (single-vessel PCI, two-vessel PCI, and three-vessel PCI) by procedure codes for analysis. The AMI patients receiving two-vessel PCI and three-vessel PCI during the index procedure were collected into the AMI cohort of immediate multivessel PCI for comparison study (Figure 1).

### Outcome Analysis

To measure the outcome, we define mortality as the end date of NHI coverage. Because NHI in Taiwan is mandatory, and its premium is paid monthly, the maximal error was limited within 1 month. All enrolled patients were followed until death or December 31, 2012, whichever occurred first. The difference between the date of admission and the end date of NHI coverage offers a valid measure of survival (17). Furthermore, other adverse cardiovascular events, including hospitalizations due to recurrent myocardial infarction and stroke, were analyzed.

### Statistical Analyses

Percentile values were used to express categorical data, which was analyzed by using the Chi-squared test. Mean and standard deviation (SD) were applied to describe continuous variables, compared by the paired *t-*test. The Cox proportional hazard regression model was applied to calculate the hazard ratio (HR) and associated 95% confidence interval (95% CIs). The Kaplan–Meier cumulative survival curves were used to analyze outcomes between the AMI patients treated by PCI at the different number of vessels and the control group and compare subgroups including types of AMI, gender, age, and other comorbidities. Comparisons of outcomes between the groups of immediate multivessel PCI and staged multivessel PCI were also performed to analyze the optimal timing of complete revascularization. The differences between the curves were tested with a log-rank test. All statistical analyses were performed using SAS version 9.4 (SAS Institute, Inc., Cary, NC). Two-sided statistical tests with *P* < 0.05 were considered statistically significant.

## Results

### Descriptive Characteristics of the Study Group

A total of 186,112 patients with AMI were enrolled in our study, with 73,148 (39.3%) patients of STEMI (ST-elevation myocardial infarction) and 112,964 (60.7%) patients of NSTEMI (non-ST-elevation myocardial infarction), respectively. Patient selection reflected the real distribution of AMI treatment in Taiwan. The demographic and clinical characteristics of AMI patients eligible for analysis are shown in Tables 1, 2. Male prevalence was high (128,209/186,112, 68.9%) in these populations, as well as the prevalence of other traditional cardiovascular risk factors. PCI was performed in 78,699 (42.3%) patients with AMI, mostly done at single vessel (62605/78699, 80.0%), and only nearly one-third of patients with NSTEMI underwent PCI. Among AMI patients who did not receive PCI, the result revealed higher proportions of elderly and comorbidities. When comparing the group of immediate multivessel PCI to the staged multivessel PCI group, higher proportions of NSTEMI and higher prescription rates of evidence-based medications were observed in the former group.

**Table 1:** Characteristics of hospitalized patients with first AMI, managed with different PCI strategies.

	**No PCI *N =* 107,413**	**Single-vessel PCI *N =* 62,605**	**Two-vessel PCI *N =* 12,662**	**Three-vessel PCI *N =* 1,516**	***P***
	**n (%)**	**n (%)**	**n (%)**	**n (%)**	**4 groups**
**Male**	67,550 (62.89)	48,749 (77.87)	9,323 (73.63%)	1,086 (71.64)	<0.0001
**Age** **≧** **75**	47,352 (44.08)	14,283 (22.81)	3,698 (29.21)	487 (32.12)	<0.0001
**Type**					<0.0001
NSTEMI	76,873 (71.57)	26,009 (41.54)	8,322 (65.72)	1,049 (69.20)	
STEMI	30,540 (28.43)	36,596 (58.46)	4,340 (34.28)	467 (30.80)	
**Comorbidities**					
HTN	65,795 (61.25)	34,072 (54.42)	7,960 (62.87)	990 (65.30)	<0.0001
Dyslipidemia	34,966 (32.55)	38,351 (61.26)	8,014 (63.29)	984 (64.91)	<0.0001
DM	44,225 (41.17)	21,556 (34.43)	5,667 (44.76)	740 (48.81)	<0.0001
PVD	5,320 (4.95)	1,276 (2.04)	373 (2.95)	54 (3.56)	<0.0001
ESRD	5,344 (4.98)	1,861 (2.97)	694 (5.48)	105 (6.93)	<0.0001
CVA	4,275 (3.98)	1,142 (1.82)	268 (2.12)	37 (2.44)	<0.0001
Heart failure	10,879 (10.13)	1,805 (2.88)	586 (4.63)	91 (6.00)	<0.0001
COPD	17,877 (16.64)	4,435 (7.08)	1,121 (8.85)	133 (8.77)	<0.0001
**Treatment**					
IABP	4,746 (4.42)	5,627 (8.99)	1,394 (11.01)	200 (13.19)	<0.0001
CABG	12,391 (11.54)	3,540 (5.65)	642 (5.07)	83 (5.47)	<0.0001
textbfOutcome					
Stroke	9,533 (8.88)	4,898 (7.82)	1,057 (8.35)	125 (8.25)	<0.0001
Recurrent MI	14,370 (13.38)	6,346 (10.14)	1,402 (11.07)	156 (10.29)	<0.0001
GI bleeding	6,087 (5.67)	3,084 (4.93)	713 (5.63)	85 (5.61)	<0.0001
ICH	1,853 (1.73)	825 (1.32)	142 (1.12)	29 (1.91%)	<0.0001
**Drugs**					
Any antiplatelets	82,006 (76.35)	61,780 (98.68)	12,535 (99.00)	1,508 (99.47)	<0.0001
ACEI or ARB	54,746 (50.97)	47,322 (75.59)	9,503 (75.05)	1,162 (76.65)	<0.0001
Statin	22,604 (21.04)	33,446 (53.42)	6,907 (54.55)	840 (55.41)	<0.0001
Beta blocker	44,796 (41.70)	40,083 (64.03)	8,127 (64.18)	1,011 (66.69)	<0.0001
CCB	38,436 (35.78)	16,829 (26.88)	4,717 (37.25)	551 (36.35)	<0.0001
Heparin or LMWH	63,089 (58.73)	58,688 (93.74)	11,924 (94.17)	1,438 (94.85)	<0.0001
Dopamine	22,490 (20.94)	9,045 (14.45)	1,709 (13.50)	222 (14.64)	<0.0001
Epinephrine	4,037 (3.76)	1,635 (2.61)	473 (3.74)	59 (3.89)	<0.0001
Norepinephrine	12,819 (11.93)	4,855 (7.75)	1,261 (9.96)	192 (12.66)	<0.0001
Atropine	4,146 (3.86)	1,317 (2.10)	231 (1.82)	44 (2.90)	<0.0001
Spironolactone	11,924 (11.10)	6,741 (10.77)	1,854 (14.64)	262 (17.28)	<0.0001
Nitrate	76,907 (71.60)	56,573 (90.36)	11,852 (93.60)	1,447 (95.45)	<0.0001
Nicorandil	8,680 (8.08)	5,654 (9.03)	1,379 (10.89)	176 (11.61)	<0.0001

*STEMI, ST-elevation myocardial infarction; NSTEMI, non-ST-elevation myocardial infarction; HTN, hypertension; DM, diabetes mellitus; PVD, peripheral vascular disease; ESRD, end-stage renal disease; CVA, cerebral vascular accident; COPD, chronic obstructive pulmonary disease; IABP, intra-aortic balloon pump; CABG, coronary artery bypass graft; MI, myocardial infarction; GI, gastrointestinal; ICH, intracerebral hemorrhage; ACEI, angiotensin-converting enzyme inhibitors; ARB, angiotensin receptor blockers; CCB, calcium channel blocker; LMWH, low molecular weight heparin*.

**Table 2:** Characteristics of hospitalized patients with first AMI and multivessel disease, managed with different PCI strategies.

	**Immediate multivessel PCI *N =* 14,178**	**Staged multivessel vessel PCI *N =* 1,916**	***P*2 groups**
	**n (%)**	**n (%)**	
**Male**	10,409 (73.42)	1,501 (78.34)	<0.0001
**Age** **≧** **75**	4,185 (29.52)	453 (23.64)	<0.0001
**Type**			<0.0001
NSTEMI	9,371 (66.10)	711 (37.11)	
STEMI	4,807 (33.90)	1,205 (62.89)	
**Comorbidities**			
HTN	8,950 (63.13)	1,026 (53.55)	<0.0001
Dyslipidemia	8,998 (63.46)	1,144 (59.71)	0.0014
DM	6,407 (45.19)	736 (38.41)	<0.0001
PVD	427 (3.01)	43 (2.24)	0.0611
ESRD	799 (5.64)	52 (2.71)	<0.0001
CVA	305 (2.15)	35 (1.83)	0.3539
Heart failure	677 (4.78)	51 (2.66)	<0.0001
COPD	1,254 (8.84)	139 (7.25)	0.0202
**Treatment**			
IABP	1,594 (11.24)	231 (12.06)	0.2918
CABG	725 (5.11)	110 (5.74)	0.245
**Outcome**			
Stroke	1,182 (8.34)	206 (10.75)	0.0004
Recurrent MI	1,558 (10.99)	219 (11.43)	0.563
GI bleeding	798 (5.63)	106 (5.53)	0.8639
ICH	171 (1.21)	35 (1.83)	0.0233
**Drugs**			
Any antiplatelets	14,043 (99.05)	1,340 (69.94)	<0.0001
ACEI or ARB	10,665 (75.22)	1,103 (57.57)	<0.0001
Statin	7,747 (54.64)	857 (44.73)	<0.0001
Beta blocker	9,138 (64.45)	922 (48.12)	<0.0001
CCB	5,268 (37.16)	439 (22.91)	<0.0001
Heparin or LMWH	13,362 (94.24)	1,308 (68.27)	<0.0001
Dopamine	1,931 (13.62)	297 (15.50)	<0.0252
Epinephrine	532 (3.75)	92 (4.80)	<0.0255
Norepinephrine	1,453 (10.25)	278 (14.51)	<0.0001
Atropine	275 (1.94)	36 (1.88)	0.8562
Spironolactone	2,116 (14.92)	292 (15.24)	0.7163
Nitrate	13,299 (93.80)	1,281 (66.86)	<0.0001
Nicorandil	1,555 (10.97)	182 (9.50)	0.0518

*STEMI, ST-elevation myocardial infarction; NSTEMI, non-ST-elevation myocardial infarction; HTN, hypertension; DM, diabetes mellitus; PVD, peripheral vascular disease; ESRD, end-stage renal disease; CVA, cerebral vascular accident; COPD, chronic obstructive pulmonary disease; IABP, intra-aortic balloon pump; CABG, coronary artery bypass graft; MI, myocardial infarction; GI, gastrointestinal; ICH, intracerebral hemorrhage; ACEI, angiotensin-converting enzyme inhibitors; ARB, angiotensin receptor blockers; CCB, calcium channel blocker; LMWH, low molecular weight heparin*.

### Survival Analysis

**Figure 2:**
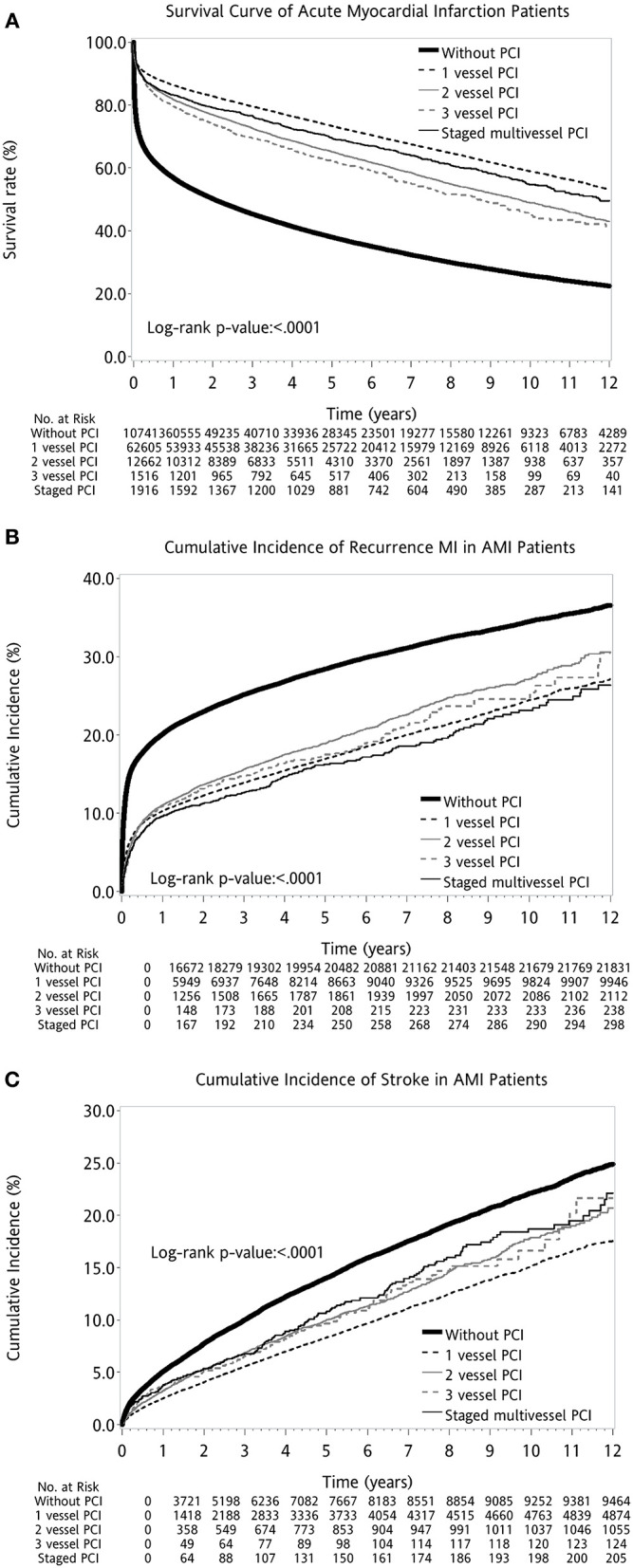
Kaplan–Meier survival curves for different outcome measures after first AMI: **(A)** Survival. **(B)** Recurrent MI. **(C)** Stroke. AMI, acute myocardial infarction; PCI, percutaneous coronary intervention.

**Figure 3:**
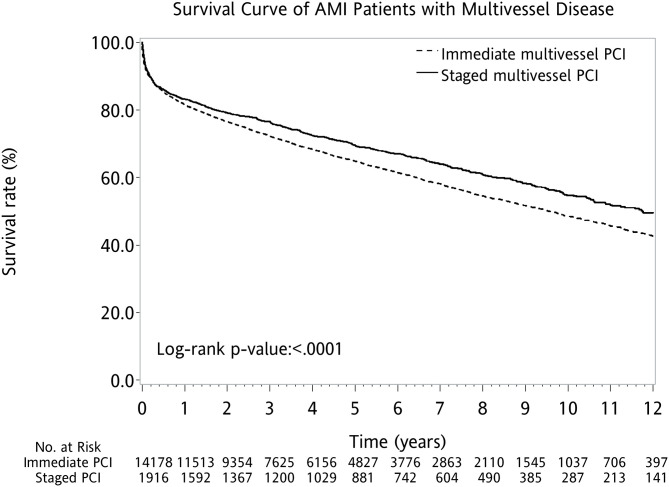
Kaplan–Meier survival curves for survival after first AMI with multivessel disease. AMI, acute myocardial infarction; PCI, percutaneous coronary intervention.

Overall, the AMI patients without PCI had worse outcomes when compared with AMI patients with PCI. The AMI cohort with single-vessel PCI had the highest survival. Among those receiving PCI for multivessel disease, the 12-year survival rate was higher for the staged multivessel PCI group, followed by patients with three-vessel PCI or two-vessel PCI (log-rank *P* < 0.001; Figure 2A). When directly comparing AMI patients with immediate multivessel PCI (two-vessel PCI and three-vessel PCI) to the cohort of staged multivessel PCI, the result also showed that the latter had better survival (log-rank *P* < 0.001; Figure 3).

When examining other long-term adverse cardiovascular events, AMI patients with staged multivessel PCI had less recurrence of myocardial infarctions but a higher incidence of stroke than other PCI strategies (log-rank *P* < 0.001; Figures 2B,C).

The AMI patients were also divided by different categories, including AMI type, gender, age, comorbidities, and intra-aortic balloon pump (IABP) placement. The Kaplan–Meier survival curves all demonstrated similar trends, with a worse outcome for two-vessel PCI or three-vessel PCI (Supplementary Figures 1–3).

The Cox proportional hazard regression analysis indicated HR for mortality of AMI patients in different variables (Table 3), with the result of the Forrest plot shown in Supplementary Figure 4. When focusing on the AMI with multivessel disease, the Cox analysis revealed that immediate multivessel PCI was associated with higher mortality risks (HR = 1.90, 95% CI: 1.73–2.10) in comparison with staged multivessel PCI during the same hospitalization, irrespective of the type of AMI (Table 4).

**Table 3:** Cox proportional hazards regression analysis for hospitalized patients with first AMI and subgroup analysis of different AMI types.

**Variables**	**All (*N =* 184,196)**		**STEMI patients (*N =* 71,943)**		**NSTEMI patients (*N =* 112,253)**	
	**HR (95% CI)**	***P-*value**	**HR (95% CI)**	***P-*value**	**HR (95% CI)**	***P-*value**
**PCI**
1 vessel vs. No PCI	0.56 (0.55–0.57)	<0.0001	0.60 (0.59–0.62)	<0.0001	0.57 (0.56–0.59)	<0.0001
2 vessels vs. No PCI	0.66 (0.64–0.68)	<0.0001	0.69 (0.65–0.73)	<0.0001	0.65 (0.62–0.67)	<0.0001
3 vessels vs. No PCI	0.70 (0.64–0.76)	<0.0001	0.70 (0.60–0.82)	<0.0001	0.69 (0.63–0.76)	<0.0001
2 vessels vs. 1 vessel	1.18 (1.14–1.22)	<0.0001	1.15 (1.09–1.21)	<0.0001	1.13 (1.09–1.18)	<0.0001
3 vessels vs. 1 vessel	1.25 (1.15–1.35)	<0.0001	1.17 (1.00–1.36)	0.0458	1.21 (1.10–1.34)	<0.0001
3 vessels vs. 2 vessels	1.06 (0.97–1.15)	0.1878	1.02 (0.87–1.20)	0.8064	1.07 (0.97–1.19)	0.1717
Gender (Male)	0.91 (0.90–0.92)	<0.0001	0.80 (0.78–0.82)	<0.0001	0.97 (0.96–0.99)	<0.0001
Age (≧75)	2.57 (2.53–2.60)	<0.0001	2.91 (2.84–2.98)	<0.0001	2.36 (2.32–2.40)	<0.0001
**Comorbidities**						
HTN	1.06 (1.05–1.08)	<0.0001	1.10 (1.07–1.13)	<0.0001	1.02 (1.00–1.04)	<0.0001
DM	1.43 (1.41–1.45)	<0.0001	1.50 (1.47–1.54)	<0.0001	1.39 (1.36–1.41)	<0.0001
PVD	1.54 (1.50–1.58)	<0.0001	1.63 (1.54–1.73)	<0.0001	1.50 (1.45–1.55)	<0.0001
HF	1.37 (1.34–1.40)	<0.0001	1.47 (1.40–1.54)	<0.0001	1.33 (1.30–1.36)	<0.0001
ESRD	1.89 (1.84–1.94)	<0.0001	2.29 (2.16–2.44)	<0.0001	1.76 (1.71–1.81)	<0.0001
CVA	1.28 (1.24–1.32)	<0.0001	1.34 (1.26–1.42)	<0.0001	1.24 (1.19–1.28)	<0.0001
COPD	1.28 (1.26–1.30)	<0.0001	1.41 (1.37–1.46)	<0.0001	1.23 (1.20–1.25)	<0.0001
**Medications**						
Any antiplatelet drugs	0.69 (0.68–0.71)	<0.0001	0.80 (0.77–0.83)	<0.0001	0.67 (0.66–0.69)	<0.0001
ACEI or ARB	0.77 (0.76–0.78)	<0.0001	0.77 (0.75–0.79)	<0.0001	0.78 (0.76–0.79)	<0.0001
Statin	0.71 (0.69–0.72)	<0.0001	0.68 (0.66–0.70)	<0.0001	0.71 (0.70–0.73)	<0.0001
Beta blocker	0.82 (0.81–0.83)	<0.0001	0.80 (0.78–0.82)	<0.0001	0.83 (0.82–0.85)	<0.0001

*STEMI, ST-elevation myocardial infarction; NSTEMI, non-ST-elevation myocardial infarction; PCI, percutaneous coronary intervention; HTN, hypertension; DM, diabetes mellitus; PVD, peripheral vascular disease; HF, heart failure; ESRD, end-stage renal disease; CVA, cerebrovascular accident; COPD, chronic obstructive pulmonary disease; ACEI, angiotensin-converting enzyme inhibitors; ARB, angiotensin receptor blockers*.

**Table 4:** Cox proportional hazards regression analysis for AMI patients with multivessel disease and subgroup analysis of different AMI type.

**Variables**	**All (*N =* 16,094)**		**STEMI patients (*N =* 6,012)**		**NSTEMI patients (*N =* 10,082)**	
	**HR (95% CI)**	***P-*value**	**HR (95% CI)**	***P-*value**	**HR (95% CI)**	***P-*value**
**PCI**						
Immediate multivessel PCI vs. Staged multivessel PCI	1.90 (1.73–2.10)	<0.0001	2.28 (2.00–2.61)	<0.0001	1.48 (1.29–1.70)	<0.0001
Gender (Male)	0.79 (0.77–0.81)	<0.0001	0.76 (0.73–0.79)	<0.0001	0.84 (0.80–0.87)	<0.0001
Age (≧75)	3.04 (2.96–3.13)	<0.0001	3.28 (3.15–3.41)	<0.0001	2.74 (2.64–2.85)	<0.0001
**Comorbidities**						
HTN	1.11 (1.08–1.14)	<0.0001	1.11 (1.07–1.15)	<0.0001	1.06 (1.02–1.11)	0.0049
DM	1.60 (1.56–1.65)	<0.0001	1.59 (1.53–1.65)	<0.0001	1.60 (1.54–1.66)	<0.0001
PVD	1.79 (1.69–1.91)	<0.0001	1.79 (1.62–1.99)	<0.0001	1.77 (1.64–1.91)	<0.0001
HF	1.74 (1.66–1.83)	<0.0001	1.79 (1.64–1.96)	<0.0001	1.70 (1.60–1.81)	<0.0001
ESRD	2.41 (2.29–2.53)	<0.0001	2.78 (2.53–3.06)	<0.0001	2.18 (2.05–2.32)	<0.0001
CVA	1.47 (1.36–1.58)	<0.0001	1.59 (1.42–1.77)	<0.0001	1.34 (1.20–1.49)	<0.0001
COPD	1.63 (1.57–1.69)	<0.0001	1.74 (1.64–1.84)	<0.0001	1.51 (1.44–1.59)	<0.0001
**Medications**						
Any antiplatelet drugs	0.42 (0.39–0.46)	<0.0001	0.38 (0.34–0.43)	<0.0001	0.48 (0.42–0.54)	<0.0001
ACEI or ARB	0.75 (0.73–0.77)	<0.0001	0.74 (0.71–0.77)	<0.0001	0.76 (0.73–0.79)	<0.0001
Statin	0.69 (0.67–0.71)	<0.0001	0.69 (0.66–0.72)	<0.0001	0.68 (0.65–0.70)	<0.0001
Beta blocker	0.80 (0.78–0.82)	<0.0001	0.78 (0.75–0.81)	<0.0001	0.81 (0.78–0.84)	<0.0001

*STEMI, ST-elevation myocardial infarction; NSTEMI, non-ST-elevation myocardial infarction; PCI, percutaneous coronary intervention; HTN, hypertension; DM, diabetes mellitus; PVD, peripheral vascular disease; HF, heart failure; ESRD, end-stage renal disease; CVA, cerebrovascular accident; COPD, chronic obstructive pulmonary disease; ACEI, angiotensin-converting enzyme inhibitors; ARB, angiotensin receptor blockers*.

## Discussion

This study, including over 180,000 patients with a 12-year follow-up, provides evidence on the importance of the timing of complete revascularization in AMI patients with multivessel disease. Performing multivessel intervention at the time of index PCI was associated with worse survival when compared with staged multivessel PCI during the index admission, regardless of STEMI or NSTEMI. Staged multivessel PCI also resulted in less recurrence of myocardial infarctions when compared to other PCI strategies, with the attention of increased events of stroke.

Several earlier studies have examined the impact of multivessel PCI on AMI patients with various inclusion criteria, timing of non-culprit vessel PCI, statisticalheterogeneity, endpoints, and conclusions. Because of conflicting results and lack of robust evidence, there is divergent clinical practice (10–16). Current guidelines suggested that PCI of non-infarct arteries should be considered in STEMI patients with the multivessel disease before hospital discharge, either at the time of primary PCI or as a planned staged procedure (18–20). This recommendation was based on serial randomized controlled trials with the results trending toward the benefits of complete revascularization, although the effects were driven mainly by a difference in the rate of repeat revascularization (11–13). Recently, the COMPLETE (Complete vs. Culprit-Only Revascularization Strategies to Treat Multivessel Disease after Early Percutaneous Coronary Intervention for STEMI) trial, a large randomized trial, found that complete revascularization by staged PCI resulted in a significant reduction of cardiovascular death or new myocardial infarction when compared with culprit-only PCI (14). However, the optimal timing for revascularization of non-infarct-related coronary arteries remained unclear since immediate PCI of non-culprit lesions in the index procedure was not allowed in the COMPLETE trial. Our research revealed that when compared with staged multivessel PCI in STEMI patients, immediate multivessel PCI during the index procedure resulted in a worse outcome. Due to higher prothrombotic and proinflammatory status in STEMI, immediate multivessel PCI for non-infarct vessels may carry a higher risk of abrupt vessel closure or stent thrombosis. Consistent with previous observational studies, our results suggested that complete revascularization during primary PCI for STEMI may still not be justified (21–25).

In contrast to the STEMI setting, there are much fewer trials examining the role of different PCI strategies in patients with NSTEMI. Determining the culprit artery in NSTEMI is not always as apparent as in STEMI. This may partly explain previous observational studies suggesting that patients with NSTEMI and multivessel disease may benefit from intervention with a complete revascularization strategy (26–30). Recent data from the British Cardiac Intervention Society PCI database also showed lower mortality rates for NSTEMI patients undergoing complete revascularization in immediate multivessel PCI than for patients with culprit-lesion-only PCI. Notably, an initial increase in in-hospital mortality was observed in the group of immediate multivessel PCI (31). This raises the concern that the complete revascularization by immediate multivessel PCI may carry several potential disadvantages, such as prolonged radiation exposure, risk of acute kidney injury, and volume overload (7). The severity of stenosis at non-infarct vessels may also be acutely exaggerated in the background of catecholamine-mediated vasoconstriction (8). Furthermore, this study did not provide information about immediate vs. staged revascularization strategy. The SMILE [Impact of Different Treatment in Multivessel Non-ST-elevation Myocardial Infarction [NSTEMI] Patients] trial was the only randomized trial addressing this issue. The result revealed that complete revascularization with immediate multivessel PCI resulted in fewer major adverse cardiovascular and cerebrovascular events than the staged revascularization strategy (15). Recently, another national registry data from Korea found that complete revascularization reduced major adverse cardiac events compared with culprit-only PCI. However, there was no benefit with either immediate multivessel revascularization or staged multivessel revascularization (16). After risk stratification by the Global Registry of Acute Coronary Events (GRACE) score, immediate multivessel PCI's potential benefit was only found in low-to-intermediate risk NSTEMI patients. In contrast, our result revealed the potential survival benefit of staged multivessel PCI, since the SMILE trial excluded the patients with cardiogenic shock, chronic total occlusions, and previous CABG surgery, limiting the generalizability of the results. It is also known that NSTEMI patients with cardiogenic shock have worse outcomes compared to those with STEMI and cardiogenic shock (32). Interventions of the non-culprit vessel may aggravate hemodynamic instability and jeopardize the viable myocardium in the setting of AMI. The higher proportions of cardiogenic shock requiring IABP and vasopressors in our study populations may contribute to the discrepancies. Another cause of divergence is possibly the higher use of fractional flow reserve (FFR) in the Smile trial, which was nearly 10 times the clinical practice setting of AMI (15, 33). Further studies are warranted to assess the role of FFR in NSTEMI.

Another potentially important finding in our study is that the multivessel intervention was associated with higher risks of stroke. Previous research has confirmed that the extent of coronary artery disease was independently related to the presence of carotid stenosis (34). Screening of carotid artery stenosis should be considered, especially in older patients with multivessel coronary artery disease. Our result was also partially attributed to increased complexity during multivessel PCI, such as chronic total occlusion intervention or high-risk PCI with IABP support (35). Compared to transfemoral intervention, transradial intervention brought a benefit of reduced risk of periprocedural stroke and thus recommended as preferred access for PCI (36).

The strength of this retrospective study lies in the use of population-based data from NHI, which covered nearly all citizens of Taiwan and provided data with large patient numbers and long periods of time. A large sample size reduces the variability in sampling statistics. NHI also ensured that patients could receive appropriate management, regardless of socioeconomic status. Thus, it would not affect the physician's or patient's decision about invasive interventions. The prescribed medications and invasive procedures would also be scrutinized by peer review regularly, which ensured the accuracy of diagnosis and treatment indications. Besides, most previous studies were from western countries; whether the results could be extrapolated to Asian populations was unclear. Our study offers information on Asian patients with AMI in clinical practice. Unlike selected low-risk populations in randomized trials, this is a real-world analysis of unselected patients with AMI.

Our study has a few limitations. First, this retrospective cohort trial has the limitations of an observational study and the potential bias and unmeasured confounding that cannot be excluded. Second, clinical values such as cardiac biomarkers, left ventricular ejection fraction, and Killip classification were unavailable. Similarly, the individual differences in coronary artery anatomy, lesion characteristics, and reperfusion status were not shown. Although potential bias remains, the accuracy of NHIRD as a valid resource for research of cardiovascular disease had been confirmed in a previous study (37). These validations include diagnosis or medications and PCI and stenting, with a positive predictive value of more than 0.9 based on the information from the discharge notes of medical records from a tertiary medical center (37). Third, the staged multivessel PCI group in our study did not include the patients receiving staged procedures in further hospitalizations after first AMI. The optimal timing of staged PCI procedures was not investigated. Finally, during the past decade, there has been a significant increase in the use of intravascular ultrasound, optical coherence tomography, FFR, and the newer generation of drug-eluting stents. The technical aspects of management of chronic total occlusion also improved. Our study started in 2000, when related medical equipment was not covered by the National Health Insurance and not routinely used. The influences of these factors might be underestimated in our study and should be taken into account for future research. Despite this, our study is the largest study evaluating different intervention strategies in Asian patients with first AMI.

## Conclusion

This nationwide cohort study found that multivessel PCI during the index procedure was independently associated with a higher risk of long-term mortality than staged multivessel PCI. This finding was observed both in patients with STEMI and in patients with NSTEMI. The incidence of stroke was higher in the AMI patients with multivessel PCI during hospitalization, which pursues further study for preventive and management strategy. Although there are limitations of the observation study, our research has a large sample size and most extended reported follow-up, which is powered enough to reflect everyday clinical practice and its impact on AMI patients' long-term outcomes.

## Data Availability Statement

The raw data supporting the conclusions of this article will be made available by the authors, without undue reservation.

## Ethics Statement

We do not need to obtain informed consent from the study patients because the NHI data consists of de-identified secondary data for research purposes. The study protocol was reviewed and approved by the Human Research Committee of Kaohsiung Veterans General Hospital.

## Author Contributions

W-CH was responsible for the study concept and design. CH, C-HChi, and C-HCha analyzed the data. C-CC, F-YK, and G-YM interpreted the data. E-SL drafted the manuscript. All the authors revised and approved the manuscript.

## Conflict of Interest

The authors declare that the research was conducted in the absence of any commercial or financial relationships that could be construed as a potential conflict of interest.

## Supplementary Material

Supplementary Figure 1 Kaplan–Meier survival curves for survival after first AMI according to the type of AMI: **(A)** comparison of survival between STEMI patients managed with 1-vessel PCI, 2-vessel PCI, 3-vessel PCI, and without PCI treatment; **(B)** comparison of survival between NSTEMI patients managed with 1-vessel PCI, 2-vessel PCI, 3-vessel PCI, and without PCI treatment. AMI, acute myocardial infarction; PCI, percutaneous coronary intervention; STEMI, ST elevation myocardial infarction; NSTEMI, non-ST-elevation myocardial infarction.

Supplementary Figure 2 Kaplan–Meier survival curves for survival after first AMI according to age and gender. **(A)** Comparison of survival between male patients managed with 1-vessel PCI, 2-vessel PCI, 3-vessel PCI, and without PCI treatment; **(B)** comparison of survival between female patients managed with 1-vessel PCI, 2-vessel PCI, 3-vessel PCI, and without PCI treatment; **(C)** comparison of survival between patients ≧75 years of age managed with 1-vessel PCI, 2-vessel PCI, 3-vessel PCI, and without PCI treatment; **(D)** comparison of survival between patients <75 years of age managed with 1-vessel PCI, 2-vessel PCI, 3-vessel PCI, and without PCI treatment. AMI, acute myocardial infarction; PCI, percutaneous coronary intervention.

Supplementary Figure 3 Kaplan–Meier survival curves for survival after first AMI in subgroup analysis of different comorbidities and IABP management. **(A)** Comparison of survival between patients with CVA managed with 1-vessel PCI, 2-vessel PCI, 3-vessel PCI, and without PCI treatment; **(B)** comparison of survival between patients without CVA managed with 1-vessel PCI, 2-vessel PCI, 3-vessel PCI, and without PCI treatment; **(C)** comparison of survival between patients with DM managed with 1-vessel PCI, 2-vessel PCI, 3-vessel PCI, and without PCI treatment; **(D)** comparison of survival between patients without DM managed with 1-vessel PCI, 2-vessel PCI, 3-vessel PCI, and without PCI treatment; **(E)** comparison of survival between patients with PVD managed with 1-vessel PCI, 2-vessel PCI, 3-vessel PCI, and without PCI treatment; **(F)** comparison of survival between patients without PVD managed with 1-vessel PCI, 2-vessel PCI, 3-vessel PCI, and without PCI treatment; **(G)** comparison of survival between patients with IABP managed with 1-vessel PCI, 2-vessel PCI, 3-vessel PCI, and without PCI treatment; **(H)** comparison of survival between patients without IABP managed with 1-vessel PCI, 2-vessel PCI, 3-vessel PCI, and without PCI treatment. AMI, acute myocardial infarction; PCI, percutaneous coronary intervention; CVA, cerebrovascular accident; DM, diabetes mellitus; PVD, peripheral vascular disease; IABP, intra-aortic balloon pump.

Supplementary Figure 4 Forest plots for the subgroup analysis of AMI patients managed with different PCI strategies. AMI, acute myocardial infarction; PCI, percutaneous coronary intervention; CVA, cerebrovascular accident; COPD, chronic obstructive pulmonary disease; ACEI, angiotensin-converting enzyme inhibitors; ARB, angiotensin receptor blockers.

## Abbreviations

ACEI: angiotensin-converting enzyme inhibitor
AMI: acute myocardial infarction
ARB: angiotensin receptor blocker
CABG: coronary artery bypass graft
CI: confidence interval
COPD: chronic obstructive pulmonary disease
CVA: cerebrovascular accident
DM: diabetes mellitus
ESRD: end-stage renal disease
FFR: fractional flow reserve
HF: heart failure
HR: hazard ratio
HTN: hypertension
IABP: intra-aortic balloon pump
NHI: National Health Insurance
NHIRD: National Health Insurance Research Database
NSTEMI: non-ST elevation myocardial infarction
PCI: percutaneous coronary intervention
PVD: peripheral vascular disease
SD: standard deviation
STEMI: ST elevation myocardial infarction.
