# Supplementary figures and images for: Comparison of Different Timing of Multivessel Intervention During Index-Hospitalization for Patients With Acute Myocardial Infarction
Source: Front Cardiovasc Med. 2021 Jun 10;8:639750. doi: 10.3389/fcvm.2021.639750 (PMC8222548; doi:10.3389/fcvm.2021.639750)

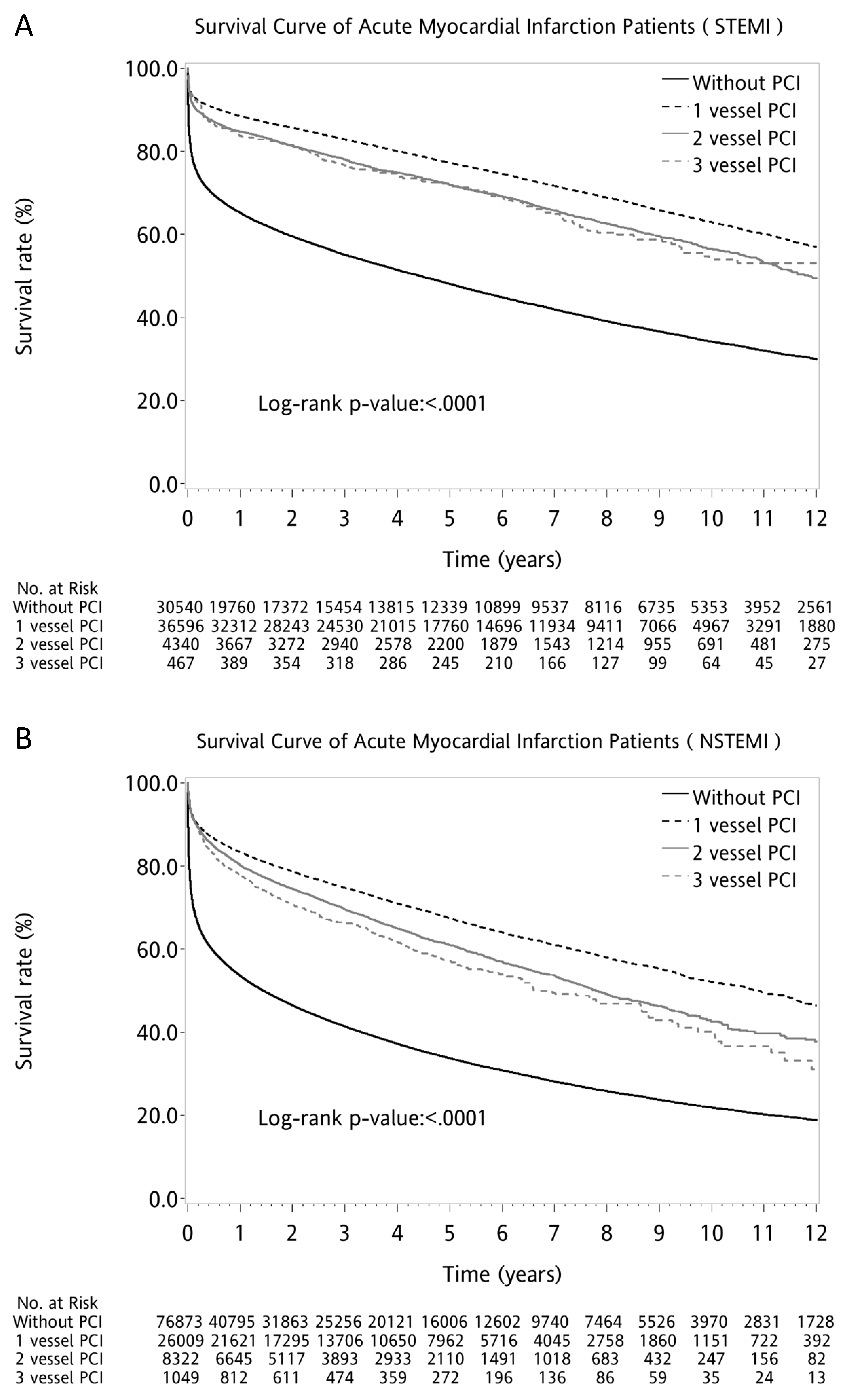

Supplement: Supplementary Figure 1 — Kaplan–Meier survival curves for survival after first AMI according to the type of AMI: (A) comparison of survival between STEMI patients managed with 1-vessel PCI, 2-vessel PCI, 3-vessel PCI, and without PCI treatment; (B) comparison of survival between NSTEMI patients managed with 1-vessel PCI, 2-vessel PCI, 3-vessel PCI, and without PCI treatment. AMI, acute myocardial infarction; PCI, percutaneous coronary intervention; STEMI, ST elevation myocardial infarction; NSTEMI, non-ST-elevation myocardial infarction. [file Image_1.TIFF]

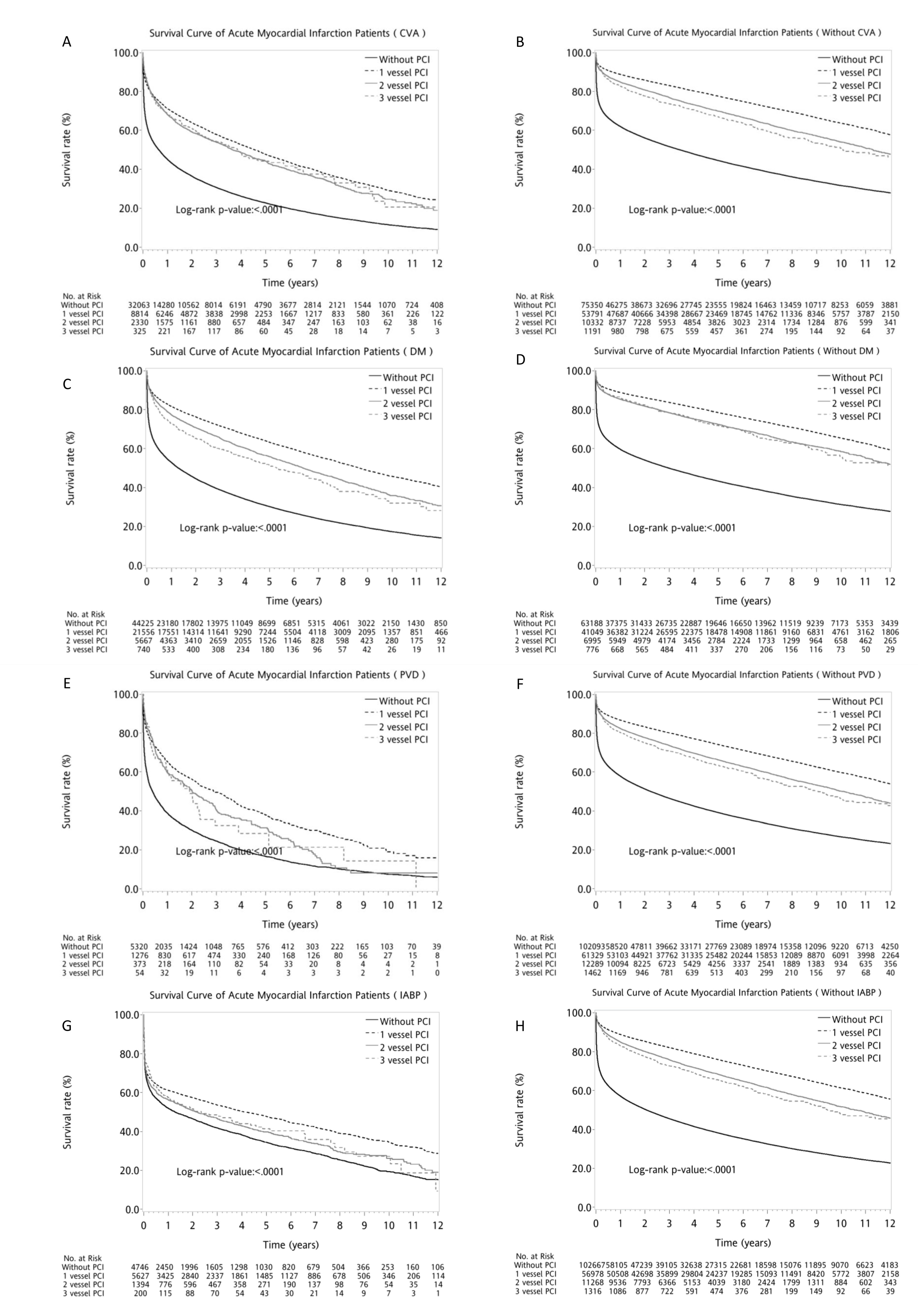

Supplement: Supplementary Figure 3 — Kaplan–Meier survival curves for survival after first AMI in subgroup analysis of different comorbidities and IABP management. (A) Comparison of survival between patients with CVA managed with 1-vessel PCI, 2-vessel PCI, 3-vessel PCI, and without PCI treatment; (B) comparison of survival between patients without CVA managed with 1-vessel PCI, 2-vessel PCI, 3-vessel PCI, and without PCI treatment; (C) comparison of survival between patients with DM managed with 1-vessel PCI, 2-vessel PCI, 3-vessel PCI, and without PCI treatment; (D) comparison of survival between patients without DM managed with 1-vessel PCI, 2-vessel PCI, 3-vessel PCI, and without PCI treatment; (E) comparison of survival between patients with PVD managed with 1-vessel PCI, 2-vessel PCI, 3-vessel PCI, and without PCI treatment; (F) comparison of survival between patients without PVD managed with 1-vessel PCI, 2-vessel PCI, 3-vessel PCI, and without PCI treatment; (G) comparison of survival between patients with IABP managed with 1-vessel PCI, 2-vessel PCI, 3-vessel PCI, and without PCI treatment; (H) comparison of survival between patients without IABP managed with 1-vessel PCI, 2-vessel PCI, 3-vessel PCI, and without PCI treatment. AMI, acute myocardial infarction; PCI, percutaneous coronary intervention; CVA, cerebrovascular accident; DM, diabetes mellitus; PVD, peripheral vascular disease; IABP, intra-aortic balloon pump. [file Image_3.TIFF]
